# Serotonin Receptors in Hippocampus

**DOI:** 10.1100/2012/823493

**Published:** 2012-05-02

**Authors:** Laura Cristina Berumen, Angelina Rodríguez, Ricardo Miledi, Guadalupe García-Alcocer

**Affiliations:** ^1^Facultad de Química, Universidad Autónoma de Querétaro, Centro Universitario S/N, Cerro de las Campanas, Querétaro 76010, Mexico; ^2^Instituto de Neurobiología, Universidad Nacional de México, Campus Juriquilla, Querétaro 76230, Mexico; ^3^Department of Neurobiology and Behaviour, University of California, Irvine, CA 92697-4550, USA

## Abstract

Serotonin is an ancient molecular signal and a recognized neurotransmitter brainwide distributed with particular presence in hippocampus. Almost all serotonin receptor subtypes are expressed in hippocampus, which implicates an intricate modulating system, considering that they can be localized as autosynaptic, presynaptic, and postsynaptic receptors, even colocalized within the same cell and being target of homo- and heterodimerization. Neurons and glia, including immune cells, integrate a functional network that uses several serotonin receptors to regulate their roles in this particular part of the limbic system.

## 1. Serotonin

**Figure 1:**
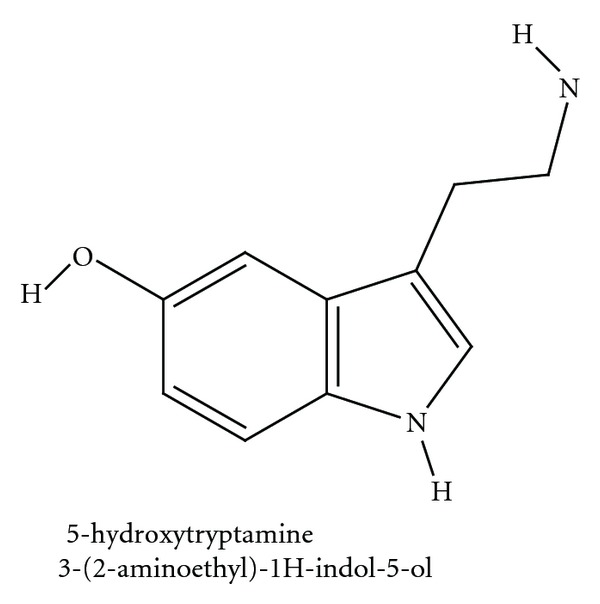
Serotonin (5-HT). Modified image from NCBI PubChem Substance Database CID 5202.

Serotonin (5-hydroxytryptamine; 5-HT), named by Rapport et al. (1948) [1], is one of the ubiquitous molecules acting as messengers, well known as a neurotransmitter and neuromodulator. Serotonin (Figure 1) is mostly found outside the central nervous system [2]; it was first identified in enterochromaffin cells and named as “enteramine” by Vialli and Erspamer in 1937 and confirmed to be the same entity with the “clotted blood” vasoconstriction effects in 1952 [3].

## 2. Serotonin as an Ancient Molecular Signal

The serotonergic system is an ancient sensor of diverse stimuli and molecular signaling in single-celled eukaryotes, plants, and animals [4–6].

**Figure 2:**
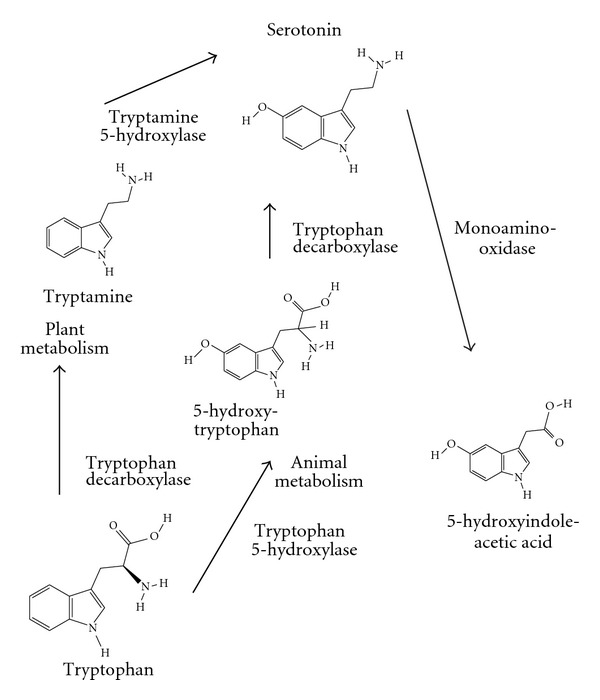
Serotonin metabolism. Tryptophan is the precursor for serotonin synthesis, with different enzymatic reactions in plant and animals [6]; hydroxylation is the rate limiting step (enzyme mediated by tryptophan hydroxylase in animals or tryptamine hydroxylase in plants), while decarboxylation is a rapid conversion by the aromatic amino acid decarboxylase (tryptophan decarboxylase). The catabolic metabolite of serotonin is 5-hydroxyindoleacetic acid, via 5-hydroxyindole acetaldehyde enzymatically converted by the membrane-bound mitochondrial flavoprotein monoamino oxidase. Modified images from NCBI PubChem Substance Database.

The regulated expression of genetic material in every cell is very important and a “regulatory lesson” learned over the years is that small metabolites are often regulatory signals to control gene expression. For “expensive” biosynthesis, as the required for the serotonin precursor tryptophan, common pathways are found in organisms that take advantage of the aromatic structures; tryptophan serves as the precursor not only of serotonin (Figure 2), but also of very important compounds as niacin in eukaryotes, indoleacetic acid in plants, and indole in bacteria. Regulatory strategies could be compatible with other metabolic goals as organisms evolved capable of obtaining tryptophan by feeding, with specific plasma membrane transporters [7, 8].

Beyond the heterotrophic theory of the very first living organisms [9], serotonin could be used as specific signal, after direct relation with tryptophan synthesis was controlled, and specific monoamine transporters that do not need the missing carboxyl group of the aminoacids [7, 10] were present; later, it acquired functions of “hormone” and growth factor, and serotonin activity as neurotransmitter was achieved at last [4]. In prenervous stages, serotonin regulates basic developmental processes from cleavage divisions after fertilization (proliferator) to morphogenetic cell movements during gastrulation (morphogen) in sea urchin [11]. Presence of serotonin and its metabolite 5-hydroxyindoleacetic acid in unicellular ciliate *Tetrahymena pyriformis* [12] and increasing RNA production in the 5-HT stimulated protozoa [13] suggested an active biogenic amine system with relevant functions; interaction with GTPases might represent some of the earlier functions of serotonin (and biogenic amines) before it could be vesiculated and its exocytosis could be regulated for metazoan serotonergic systems [14, 15].

## 3. Serotonin as a Regulatory Molecule in Animals

This happy hormone, as recalled by Dr. Barnes [16], plays a modulatory role in almost every physiological function and is involved in many biological processes [2, 17]; furthermore, the three related metabolites, 5HT, tryptophan, and melatonin, are important regulators of feed intake, reproduction, immunity, neurological function, and antistress responses [18].

Serotonin is involved in natural reward-related physiology and behaviour, from feeding to sexual activity [19] with many actions correlated to the involved location (cellular-tissue-organ concentration) and the different signaling can also be associated with its more than fourteen receptor subtypes, regulating physiological processes through different, even opposing mechanisms; these indoleamine effects include also serotonylation and interaction with GTPases [2, 14, 15]. Serotonin influences body temperature, breathing rhythms (respiratory system), heart rate (cardiovascular function in general), eating and bowel motility (gastrointestinal system), ejaculatory latency and bladder control, muscle contraction/relaxation and locomotion, sleep, arousal, pain and sensory perception, emotions, and cognition [2, 5, 20] with a well-known signaling role in immune cells [21].

## 4. Serotonin in Central Nervous System

Serotonergic neurons, first discovered in the brainstem by Dahlström and Fuxe in 1964 [22], release 5-HT throughout the CNS [23, 24] as expected after the brain serotonin discovery [25]. 5-HT cell bodies are mainly localized in the raphe nuclei with their axons innervating almost every brain region [17]. The hippocampus is a principal target of serotonergic afferents along with all the limbic system [26].

The serotonin projections to hippocampus stem in a topographic order from the midbrain dorsal and median raphe nuclei [27–29]. The rat ventral hippocampus receive moderately dense projections from the caudal dorsal raphe and essentially none from the rostral dorsal raphe, with fine serotonergic axons and small varicosities widely distributed throughout the hippocampus. Furthermore, beaded serotonergic axons with large, spherical varicosities are also found in hippocampus; median raphe nucleus predominantly innervate the stratum lacunosum moleculare of the CA1 and CA3 regions and the dentate hilus [26, 28, 30, 31]. The density of serotonergic axons is highest in CA3, lower in dentate gyrus and lowest in CA1 [26, 30]. Almost all subtypes of serotonin receptors are expressed in hippocampus during ontogeny, so the regulation of the serotonergic system is more than complex [32, 33].

## 5. Serotonin Receptors

Heterogeneity in serotonin receptors was established by the late 1950s, with Gaddum and Picarelli [34] proposing two tryptamine receptors in the guinea-pig ileum: M and D, blocked with morphine and dibenzyline, respectively; binding to serotonin receptors was also studied with [^3^H] 5-HT and [^3^H] LSD [35, 36] and more than twenty years later a new classification was proposed by Peroutka and Snyder (1979): 5-HT1 and 5-HT2 receptors based on radioligand binding techniques ([^3^H] 5-HT, [^3^H] LSD and [^3^H] spiroperidol) [37].

With the use of specific radiolabelled ligands, there was a new classification [38] proposing 5-HT3 receptors although 5-HT1-like receptors were still considered a heterogeneous entity. Others tried to adjust the new information and finally, with the advances in molecular biology, the serotonin receptors were cloned, finding more than three subtypes. The Serotonin Club Receptor Nomenclature Committee (SCRNC), reporting directly to the IUPHAR Committee for Receptor Nomenclature, described a new classification of 5-HT receptors [39]. This classification was based in different operational (selective agonists, antagonists, and ligand-binding affinities), structural (molecular structure), and transductional (intracellular transduction mechanisms) criteria.

**Figure 3:**
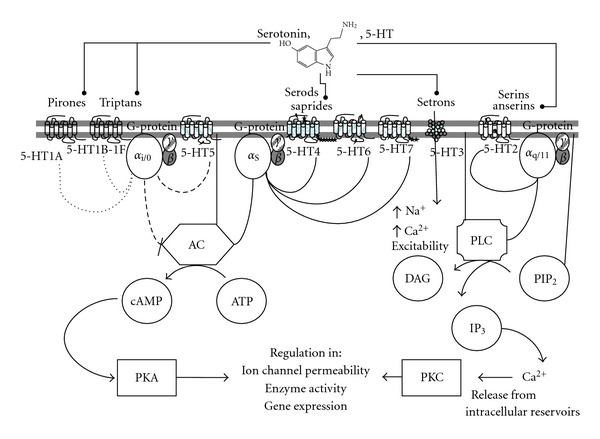
Serotonin main signaling pathways. 5-HT or agonists/antagonists for each receptor (•) interact in the extracellular side and the conformational changes of 5-HTRs modify the activity of specific intracellular enzymes, which in time modify other targets state to provoke different cellular responses [43]. G-protein *βγ* pathways are not represented in the figure. All of the serotonin receptor subtypes are represented for a hippocampal pyramidal cell, as reported, but subpopulations of these neurons might differentially express 5-HT receptors. AC, adenylate cyclase; PLC, phospholipase C. The 7TMD images of each subtype receptor are represented with the defined number of exons that code for the mature protein [44]; putative intron location in correspondent pre-mRNA is marked by a lightning symbol (*↯*), and alternative splicing sites are marked with stars (*⋆⋆⋆*).

Serotonergic receptors (Figure 3) were grouped in seven classes 5-HT_1–7_, all of them belonging to the G-protein-coupled receptor (GPCR) superfamily [40], except 5-HT_3_ which is a ligand-gated ion channel that belongs to the nicotinic acetylcholine receptor superfamily: cystein-loop transmitter gated superfamily which constitutes heteropentamers [5, 41, 42]. Particularly, subindex for the different receptors were arranged and the former 5-HT1C was renamed as 5-HT_2C_, for its transductional properties and molecular structure [39]. In the paper, subscript will be used for 5-HT subtype receptors after SCRNC, and normal line of type for previous findings in subtype receptor will be written.

## 6. Ion Channel Serotonin Receptor

The 5-HT_3_ receptor is a cation-selective ion channel which activation evokes neuronal excitation and neurotransmitter release. There are two well-recognized genes encoding A and B subunits, but additional C, D, and E genes expand the diversity to heterooligomer formation of the pentameric channel [45]. The different composition might reflect distinct pharmacology and relevance to their function representing each one a different subtype of receptor. These subunits can interact with other members of the Cys-loop superfamily, regarding the previous “M”-type serotonin of Gaddum and Picarrelli classification [46].

## 7. Metabotropic Serotonin Receptors

The seven transmembrane domain (7TMD) serotonin receptors belong to the “type A” family of GPCR, rhodopsin-like receptors, grouped by Fredricksson et al. (2003) in the amine receptor cluster [47]. They display a heterogeneous phylogenetic pattern with 5-HT_2_ forming one group and 5-HT_1B-1F_ forming another group; the rest of 5-HT receptor subtypes can be related with other biogenic amine receptors clusters. In other classification [48], 7TMD 5-HT receptors can be grouped in type 1 family that contains GPCRs for small ligands binding in a cavity formed by TM-III to TM VI [49].

The 7TMD serotonin receptors are coupled to different G proteins. The 5-HT_1_ receptors couple to G*α*_i_/G*α*_o_ proteins; the 5-HT_2_ receptors couple to G*α*_q_ proteins; the 5-HT_4_, 5-HT_6_ and 5-HT_7_ receptors couple to G*α*_s_ proteins, and the 5-HT_5_ receptors are related to G*α*_i_/G*α*_o_ proteins [44].

**Figure 4:**
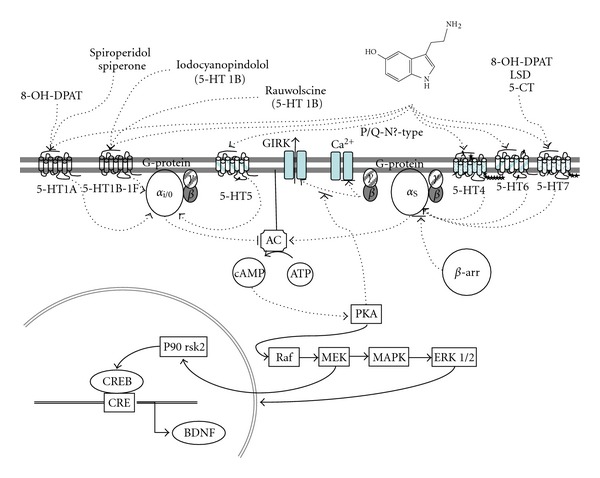
cAMP signaling pathways. Serotonin receptors 5-HT_1_ and 5-HT_5_ interact with *α*_i/0_ G-protein inhibiting the formation of cyclic adenylate monophosphate (cAMP) by adenylate cyclase (AC), while 5-HT_4_, 5-HT_6_, and 5-HT_7_ activate AC by means of *α*_S_ G-protein. *βγ* subunits of G-protein may interact in other signaling pathways, for example, modulating GIRK or calcium voltage gated channels. Representation of *β*-arrestin (*β*-arr) is made to indicate other signaling pathways. Traditional ligands to study different subtype receptors are written in the extracellular zone; note that 5-HT_7_ may bind the traditional ligand for 5-HT_1A_ as well as LSD (5-HT_2_ ligand).

Activation of G*α*_s_ coupled receptors (Figure 4) leads to the stimulation of adenylyl cyclases elevating cyclic AMP (cAMP), which as a second messenger interacts with other proteins including ion channels and activating the protein kinase A (PKA). This phosphorylating enzyme also activates cAMP-responsive transcription factors like CREB modifying gene expression. The interaction with other exchange proteins directly activated by cAMP leads to alternative signaling cascades besides the classical PKA. The interaction with G*α*_i_ leads to inhibition of adenylyl cyclases, decreasing production of cAMP [5].

**Figure 5:**
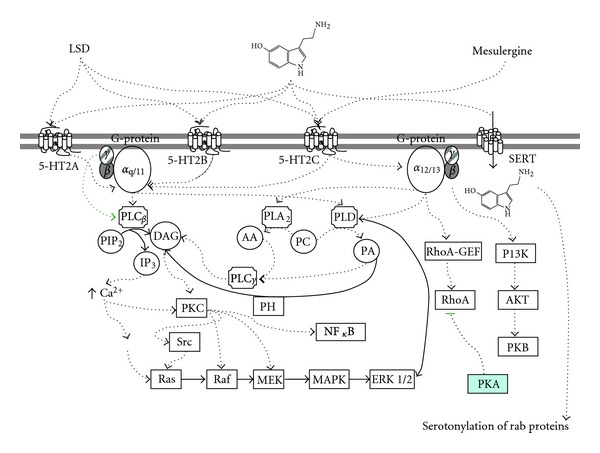
5-HT_2_ receptors signaling. Main pathways of intracellular signaling for these serotonine receptors subtype involve rupture of membrane phospholipids, particularly with phospholipase C (PLC) producing diacylglycerol (DAG) and inositol 1,4,5-trisphosphate (IP_3_) from phosphatidylinositol 4,5-bisphosphate (PIP_2_). These second messengers activate protein kinase C (PKC) which in time may activate the extracellular signal-regulated kinases 1 and 2 (ERK1/2) [50]. Phospholipase A2 is eventually activated producing arachidonic acid (AA) from phosphatidylcholine (PC), or phosphatidic acid (PA) by means of phospholipase D (PLD) [44, 50]. SERT is included in the diagram, coexisting in astrocytes for example, to emphasize the intracellular participation of serotonin itself [14]. Other pathways including (Rho-GEF) and (PI3K) are shown [51]. MEK, mitogen-activated protein kinase; PH, phosphohydrolase enzyme; PKA, protein kinase A-relation to cAMP pathways; SERT, serotonin transporter.

The activation of G*α*_q/11_ coupled receptors (Figure 5) lead to the hydrolysis of membrane phosphoinositides resulting in the formation of diacyl glycerol (DAG) and inositol phosphates (IP_3_). IP_3_ can interact with the calcium reservoirs, elevating intracellular levels and activating protein kinase C [5, 50]. Serotonin receptors may also be coupled to G*α*_12/13_, mediating structural changes within the cell through activation of the Rho signaling pathway [41].

The G_*βγ*_ dimeric subunit can interact with a variety of enzymatic effectors within the cell, like their action on gated ion channels, regulation of particular isoforms of adenylyl cyclase and phospholipase C, and phosphoinositide-3-kinase isoforms (and ERK signaling) [51].

If so many receptor subtypes of serotonin make it complex to understand, plethora of activities can be found with the coupling to multiple G-proteins. There are different parameters in the activation pathway of the GPCR receptors, considering multiple states instead of the traditional two-state model of activation and forming dimers that may have distinct pharmacology with respect to activation, signaling, and internalization and the organization in microdomains at the membrane level that may affect coupling and trafficking of G-proteins [52].

Promiscuous coupling of GPCRs to G-proteins is not a surprise, and they can also signal without coupling to them; they can activate a variety of cascades by arrestin-ergic signalling, beside the original function of these proteins in terminating coupling and endocytosis [53, 54].

In brief, there are thirteen genes coding for GPCR serotonin receptors that may couple almost every G-protein in the cell membrane and probably act without coupling to them, and two recognized genes coding for the subunits of cation-selective 5-HT_3_ ligand-gated ion channel pentameric receptor.

This diversity is further complexed by the posttranslational and co/posttransductional modifications of the protein to be produced, without talking about oligomerization of the serotonin receptors and single-nucleotide polymorphisms. There are examples of this modifications in the different receptor families with alternative splicing, RNA editing, palmitoylation, glycosylation, phosphorylation, and proteolysis, to mention a few [55].

## 8. Serotonin Receptors Expression in Hippocampus

All the serotonin receptor families are remarkably expressed in hippocampus, which is part of the limbic system, a whole structure related with memory processing, emotional association with memory, judgment, affect, and motivation or the organization of planned actions [26]. The innervation of serotonergic pathways in hippocampus and the diverse expression of serotonin receptors in this brain area reflect the overall functions related to 5-HT, in particular with cognition, mood and food intake. After recognition of hippocampal serotonergic afferents by histochemical methods (fluorescence, potassium dichromate), uptake of tritiated serotonin was achieved corroborating the wide spread of 5-HT pathways [56]. Molecular biology of the specific receptors for serotonin confirmed this knowledge.

### 8.1. 5-HT_*1*_ Receptors

The hippocampus contains a high density of 5-HT_1_ sites, most of which belong to the 5-HT_1A_ subtype [39]. Before classification of serotonin receptors on the basis of their molecular biology, distinction between the receptors in this group was based on the affinities for 8-hydroxy-2-(di-n-propylamino)tetralin (8-OH-DPAT) distinguishing 5-HT1A, lysergic acid diethylamide (LSD) and mesulergine detecting 5-HT1C, later renamed as 5-HT2C, and rauwolscine for 5-HT1D receptors, for example, but findings of new receptors with affinity for these ligands may clarify error in quantitation of the former groups.

### 8.2. 5-HT_*1*A_

Fargin et al. characterized the genomic clone G-21 that corresponded to 5-HT_1A_ sequence [57]. Gozlan et al. (1983) [58] had previously reported the existence of 5-HT1—like receptors in hippocampus on the basis of the binding experiments of [^3^H] 8-OH-DPAT. In 1986, Hoyer et al. [59] and Vergé et al. [60] confirmed these results and compared binding of 5-HT1A and 5-HT1B; later characterization was performed by chromatographic analyses of the serotonin 5-HT1A receptor solubilized from the rat hippocampus [61]. Activation of somatodendritic autoreceptors diminished 5-HT synaptic transmission [62] suggesting that 5-HT1A might represent presynaptic receptors as well as postsynaptic neurotransmission in hippocampus. At cellular levels, 5-HT_1A_ receptors are located postsynaptically in pyramidal and granular neurons of the hippocampus as well as extrasynaptic structures, by studies using highly selective 5-HT_1A_ antibodies that allowed confirmation and refinement of autoradiographic results [41]. They function as somatodendritic inhibitory receptors in raphe nuclei and presynaptically in hippocampus [63]. 5-HT_1A_ has also been detected in some astrocytes, radial glia, and ependymal and endothelial cells [64].

### 8.3. 5-HT_*1*B_

Molecular cloning of rat 5-HT_1B_ receptor was performed by Voigt et al. in 1991 [65]. Previously, 5-HT1B was defined as the nonspiperone sensitive [^3^H]5-HT binding in brain [41]; localization of 5-HT1B was described with low densities in hippocampus (gyrus dentatus > *CA1* ≥ *CA3*) by affinity differences with [^3^H] 8-OH-DPAT [60] and binding studies with [^125^I]iodocyanopindolol [66]. Immunohistochemistry analysis had also shown coexpression of 5-HT_1B_ in hippocampal cells with other serotonin receptors [67]. 5-HT_1B_ receptors are responsible for the presynaptic inhibition of neurotransmission at the local synapses between axon collaterals of CA1 pyramidal cells and other CA1 pyramidal neurons and interneurons [68]. Projection neurons from hippocampus reach the bed nucleus of the stria terminalis, where presynaptic 5-HT_1B_ receptors are involved in the inhibition of glutamate transmission [69]. Furthermore, 5-HT_1B_ hippocampal GABAergic axon terminal heteroreceptors inhibit neurotransmitter release [70].

### 8.4. 5-HT_*1*D_

Hamblin and Metcalf in 1991 [71] described sequence of human 5-HT1D serotonin receptor and two genes known as 5-HT1Da and 5-HT1Db were reported [72]. It was clear later that 5-HT1Db was the homologue receptor of rat 5-HT1B, so called 5-HT_1B_. Operational profiles between the former 5-HT1Da and 5-HT1Db receptors were almost indistinguishable, and similarities are still very present [41]. 5-HT1Da remained as the homologue of rat 5-HT1D, and so-called 5-HT_1D_. 5-HT_1D_ binding sites resemble those of 5-HT_1B_ receptors in hippocampus with very low presence [41, 73]. 5-HT_1B/1D_ receptors are found at pre- and postsynaptic sites but presynaptic receptors are predominantly located on 5-HT hippocampal nerve terminals [63].

### 8.5. 5-HT_*1*E_

There is not a clear characterization of 5-HT_1E_ due to the lack of specific ligands that might differentiate this receptor subtype; furthermore, expression of 5-HT_1E_ has not been found in rodents, because there is a stop codon in the correspondent mRNA [41]. Cloning of this receptor was achieved using cDNA synthesized from monkey cortex and human hippocampal cDNA library [74] though confirming its presence in hippocampus, previously reported by the existence of a 5-HT1E subtype in human brain with findings in radioligand studies [75].

### 8.6. 5-HT_*1*F_

When 5-HT_1F_ was found [76], it was designated as 5-HT_1Eb_ due to its related pharmacological profile; 5-HT_1F_-labeling was moderate in granule cells of the dentate gyrus and hippocampal pyramidal cells in CA1–CA3, confirming its expression in hippocampus [77].

### 8.7. 5-HT_*2*_ Receptors

Receptors from this group were originally recognized by ligands like ketanserin, mesulergine, LSD, and spiperone, which were reported to have high affinities for 5-HT2 receptors compared to 5-HT1 group [78]. These receptors are coupled to phosphatidylinositol hydrolysis although some effects may involve intracellular calcium release via an independent mechanism [79]. Hoyer et al. [80] used Ketanserin binding though localizing 5-HT2 receptors recognition sites in hippocampus.

### 8.8. 5-HT_*2*A_

On the basis of the similarity in exerting the cellular effects which reflected the structural relationship with the former 5-HT1C receptor, Pritchett et al. (1988) used oligonucleotides encoding this serotonin receptor and found 5-HT_2A_ sequence [81]. Julius et al. (1990) also found an encoding sequence for 5-HT2 which was expressed in hippocampus in a 10-fold lower level than in rat cortex [82]. The 5-HT_2A_ receptor refers to the classical D receptor described by Gaddum and Picarelli in 1957 and defined later as 5-HT2 by Peroutka and Snyder in 1979 [37]. 5-HT_2A_ expression in human hippocampus was confirmed with RT-PCR technique [83]. Immunoreactivity for 5-HT_2A_ receptor in hippocampus was found primarily in the pyramidal cell layer of CA1–CA3 and in the granular layer of dentate gyrus [84]. Agonist studies with 1-(2,5-dimethoxy-4-iodophenyl)-2-aminopropane (DOI) indicate postsynaptic receptors for 5-HT_2A_ [63]; in prelimbic prefrontal cortex, most 5-HT_2A_ receptors were postsynaptically located, but presynaptic axons and varicosities locations were found [85]. Cellular localization of 5-HT_2A_ receptors in astrocytes has been found in hippocampus [86].

### 8.9. 5-HT_*2*B_

The “last” 5-HT2-like receptor subtype to be cloned was 5-HT_2B_ [87] from rat stomach fundus. The origin and comparable sequence to 5-HT1C/2 led them to designate it as 5-HT2F (for fundus) and renamed as 5-HT_2B_ after consensus of SCRNC in 1994. Cloned human 5-HT_2B_ receptors had a high degree of homology with mouse and rat receptors although with higher affinity for ketanserin and a lower affinity for yohimbine; it was found at very low presence in the whole brain [88]. Expression of 5-HT_2B_ receptors in cultured astrocytes from hippocampus with Ca^2+^ increases after stimulation with alpha-methyl 5-HT has been reported [89]. The presence of this receptor in astrocytes was verified with immunohistochemistry and *westernblot* analysis. Furthermore, microglial cell cultures expresses 5-HT_2B_ receptors, and they are involved in the regulation of inflammatory cytokine production from blood cells [90].

### 8.10. 5-HT_*2*C_

Lübbert and colleagues cloned in 1987 [91] the mouse 5-HT1C-mRNA (actually 5-HT_2C_) extracted from choroid plexus tumors; Julius et al. (1988) characterized a cDNA encoding this protein and confirmed the receptor expression in neurons of many regions of central nervous system by *in situ *hybridization and RNA blot analysis [92]. It was first identified in porcine choroid plexus on the basis of its pharmacological properties [93] and localized by autoradiographic mapping in rat [94] and human brain, particularly in hippocampus [80].

The overall distribution of 5-HT_2C_ receptor was reported by several studies with mRNA *in situ* hybridization [95–98]. The specificity of radioligand binding ([^3^H] mesulergine) was compared with *in situ* hybridization by Mengod et al. (1990), finding high signal in the pyramidal layer of the CA3 field of rostral rodent hippocampal formation, while intense hybridization was found in the strata oriens and radiatum of the caudal CA1 area and in the ventral subiculum [97]. Furthermore, Abramowski et al. (1995) compared [^3^H] mesulergine binding with specific antibody-binding in rat and human brain [99]; Clemett et al. (2000) also studied the presence of 5-HT_2C_ protein with immunohistochemistry and *western* blotting with abundant expression in rat hippocampus [100].

### 8.11. 5-HT_*3*_ Receptors

5-HT_3_ receptor belongs to the ligand-gated ion channel superfamily and corresponds to the M receptor of Gaddum and Picarelli [41, 101]; five subunits have been cloned although only 5-HT_3A_ and 5-HT_3B_ are recognized for rodents [102–106]. The various subtypes of 5-HT_3_ may well-correspond to the pentameric heterodimer assembled between all subunits and their splice variants, and also with other members of the cys-loop superfamily, like a4-nAChR nicotinic receptor [46, 107] although this association has not been detected in porcine native 5-HT3 brain receptors [108]. On the contrary, association and coimmunoprecipitation of 5-HT_3_ and P2X_2_ ATP-gated channels has been reported [109].

All subunits have been found mainly in human intestine [110]. 5-HT_3_ mRNA was found in rat hippocampus primarily on interneurons, mediating indirect inhibitory effects on pyramidal neuron populations [111]. On the contrary, 5-HT_3_ was found in human hippocampus with predominant immunoreactivity associated with pyramidal neurons in CA_2_ and CA_3_; transcripts were also identified so hippocampal cells can produce 5-HT_3A_ and 5-HT_3B_ functionally isoforms of this ion channel [112].

### 8.12. 5-HT_*4*_ Receptors

The 5-HT_4_ receptor was first described in the central nervous system [113] stimulating adenylate cyclase; with some useful radioligands, it was showed to be distributed in hippocampus. It was cloned [114] and mRNA was localized in hippocampus by *in situ* hybridization [115].

The 5-HT_4_ receptor gene is very complex and has several possible splice variants; there are at least nine receptor splice variants reported with a number of carboxy-terminal variants but no difference in affinity for agonists or antagonists [41]. There is evidence that suggests that 5-HT_4_ receptor activity enhances cognition and provides neuroprotection, particularly on hippocampal effects [116]; 5-HT_4_ receptors on hippocampal cholinergic axon terminals are neurotransmitter release facilitating [70].

### 8.13. 5-HT_*5*_ Receptors

The 5-HT_5_ receptor group consists of two members: 5-HT_5A_ and 5-HT_5B_; human 5-HT_5B_ has been described, but it fails to encode a functional protein due to the presence of stop codons in the sequence [117–119]. They still lack physiological correlation, in part for the lack of selective agonists; the transductions pathways have not been well established although negatively coupling to adenylate cyclase has been reported [41, 43, 120].

### 8.14. 5-HT_*5*A_

Cloning and distribution of 5-HT_5A_ receptor has been reported, finding high concentration in hippocampus [119, 121, 122]. Although this receptor is a well-recognized GPCR protein, the negatively coupling to adenylated cyclase is not well established [120, 123–125], and furthermore, its coupling to multiple signal transduction pathways has been reported [126]. The 5-HT_5A_ receptor is expressed predominantly by astrocytes with very weak neuronal immunoreactivity [120].

### 8.15. 5-HT_*5*B_

Cloning and distribution of 5-HT_5B_ receptor has been reported as well, finding this receptor in hippocampus [119, 127]. The levels of expression of 5-HT_5B_ mRNA in hippocampus were high, with predominant expression in CA1 pyramidal cells [128]. It is a pseudogene in man [129], and it has been proposed that the upregulation found (particularly in hippocampus) for mice 5-HT_5B_ receptor, in response of social isolation stress, might be undertaken in humans by another receptor like 5-HT_5A_ [130].

### 8.16. 5-HT_*6*_ Receptors

Ruat et al. (1993) cloned 5-HT_6_ receptor [131], starting from the sequence of rat histamine H2 receptor with two transcripts evidenced. mRNA was detected in hippocampus and in transfected COS-7 cells 5-HT_6_ receptor was positively coupled to adenylate cyclase. Hybridization signal of 5-HT_6_ mRNA was detected in CA1, CA2, and CA3 fields of hippocampus as well as in dentate gyrus [128].

### 8.17. 5-HT_*7*_ Receptors

Ruat et al. (1993) also cloned the putative 5-HT_7_ receptor and localized it at hippocampus [132]. It is differentially expressed in CA1 cells preferentially localized on the cell body but absent in interneurons [133]. The expression in the limbic areas suggests that these receptors mediate serotoninergic controls in functions like mood, learning, or neuroendocrine and vegetative behaviors. The emerging functions of hippocampus involve several neurotransmitter networks, where 5-HT_7_ receptors can be functioning. AMPA receptor-mediated transmission between CA3 and CA1 pyramidal neurons is enhanced postsynaptically by 5-HT_7_, while 5-HT_1A_ receptors inhibit this transmission both pre- and postsynaptically [134].

## 9. Serotonergic Modulation in Hippocampus

Among the various major neurotransmitter signaling, like monoaminergic, glutamatergic, and nitrergic neurotransmitter systems that might be involved in some plastic modifications of hippocampus particularly after stress exposure [135], serotonergic system is very interesting for its complexity and regulation.

Almost all pre- and postsynaptic serotonin receptors have been identified in hippocampus; furthermore, the 5-HT transporter (SERT, 5-HTT) plays a key role in serotonergic neurotransmission, and it is condition-regulated in hippocampus [136, 137]. In addition, tryptophan hydroxylase (TPH), the rate-limiting enzyme for producing serotonin, plays another key role in the regulation of this system; TPH1 and TPH2 have been found in hippocampus [138]. The other key enzyme in serotonergic system is monoamine oxidase A, responsible for 5-HT degradation [139], expressed in hippocampus as well.

Regulation of serotonin system is very important and disturbances in this matter are related to anatomical, functional and behavioural anomalies, including neurologic and psychiatric disorders as obsessive-compulsive disorder, bulimia, chronic impulsivity, obesity and drug addiction, aggression, -major- depression, suicide, anxiety, schizophrenia, mania, autism, Alzheimer's disease and also sudden infant death syndrome [43, 139–142].

The function of serotonin as neurotransmitter seems to be developed at last in evolution, and ionotropic channels are related to rapid neuronal activation, particularly in enteric nervous system [4]. Serotonin, as metabotropic effector, has been recognized as a trophic factor, particularly during development including morphogenetic activities as cell proliferation, migration and differentiation [137, 143]; during adulthood, depletion in serotonin decreases neurogenesis in the dentate gyrus [144] though 5-HT plays a critical role in the neuronal organization of the hippocampus [145].

Several metabotropic effects of serotonin have been related to brain-derived neurotrophic factor (BDNF) expression [144] and BDNF itself promotes the development and function of serotonergic neurons [140]. This kind of interaction between neurotrophic factors and neurotransmitters has been reported also with steroids; the regulation of HPA axis by serotonin and vice versa is well documented [146, 147]; sexual steroids have this intricate correlation as well [148]. The key for understanding these relationships is the existence of multiple receptors and ligand interaction for molecular signaling.

On the other hand, hippocampus-dependent memory formation uses long-term potentiation (LTP) as a pivotal role. Cross-talk between the cAMP signal transduction system and LTP has been reported, with a critical linkage between Ca^2+^ and cAMP signaling [149]. At this level, all of the serotonin receptors seem to be directly involved in the normal function of hippocampus in mood regulation and memory formation; neurogenesis is thought to be one of the involved processes for long lasting changes related to hippocampal function, particularly because dentate gyrus is one of the prominent areas of adult brain neurogenesis [150].

The 5-HT_1A_ is the most likely involved receptor in regulation of neurogenesis in the dentate gyrus [150]; it is expressed on raphe serotonin neurons as an autoreceptor [151], acting as a negative regulator of neuronal activity in presynaptic locations in hippocampus, with very important function in the balance of serotonin reservoirs. 5-HT_1A_ also inhibits neuronal firing, activating G-protein-gated inwardly rectifying potassium (GIRK) currents and inhibiting Ca^2+^ channels [44]; it is involved in the inhibition of long-term potentiation (LTP) by the inhibition of NMDA function [152].

As one of the most “important” members of serotonin receptors, 5-HT_1A_ receptor is the best characterized and its ligands are used extensively. The mutant (knockout) mice lacking this receptor exhibits enhanced anxiety-related behaviour [153, 154]. The “specific” 5-HT_1A_ ligand 8-OH-DPAT has been used to establish the roles of this receptor as trophic factor and in neurotransmission as well, but 5-HTT (SERT) recognizes this ligand and likewise modulates anxiety-related behaviour [136, 155].

The therapeutic effects of serotonin-selective reuptake inhibitors (SSRI), “specifically” acting on SERT function, are well documented, and several theories are proposed to explain the retarded actions in successfully treated patients [156–158]. SSRIs are the most widely prescribed class of antidepressants, which increases synaptic levels of 5-HT in hours or days, but exerts the therapeutic response several weeks later [159]. The increasing levels of 5-HT cause a desensitization of 5-HT_1A_ autoreceptors with a lesser inhibition caused by this receptor in raphe neurons, leading to a facilitation of 5-HT signaling [160]. There is a differential response of SSRI's desensitizing 5-HT_1A_ presynaptic or postsynaptic receptors; the specific serotonin receptor antagonist WAY 100635 also promotes differential changes in autoreceptors compared to postsynaptic 5-HT_1A_ receptors [160, 161].

SERT and 5-HT_1A_ are the most studied therapeutic targets although several serotonin receptors are involved in hippocampus activities, particularly 5-HT_4_, 5-HT_6_, and 5-HT_7_ that activate cAMP signaling increasing CREB, which may increase the expression of BDNF [150]. Furthermore, 5-HT_4_ activation may cause a faster direct activation of 5-HT neurons, increasing their firing and causing desensitization of 5-HT_1A_ [159]. 5-HT_2_ receptors involve an alternative signaling pathway to cAMP, where increasing Ca^2+^ levels is of particular importance, relying on the crosstalk between cAMP signaling and Ca^2+^-regulated adenylyl cyclases. Knockout phenotype for 5-HT_2A_ shows decreased, anxiety while the one for 5-HT_2C_ shows increased appetite, overweight, and cognitive impairment. Serotonin receptor 5-HT_2C_ is probably the most important receptor related to food intake and energy balance (satiety and obesity), with viable targeting for weight control [20].

The most representative neurotransmitter receptor for serotonin in rapid actions is the ionotropic 5-HT_3_, which is also involved in LTP modulation in hippocampus [162]. The knockout phenotype for 5-HT_3A_ has reduced pain perception and variants of the 5-HT_3A_ receptor have been associated with bipolar disorder and schizophrenia [43].

**Figure 6:**
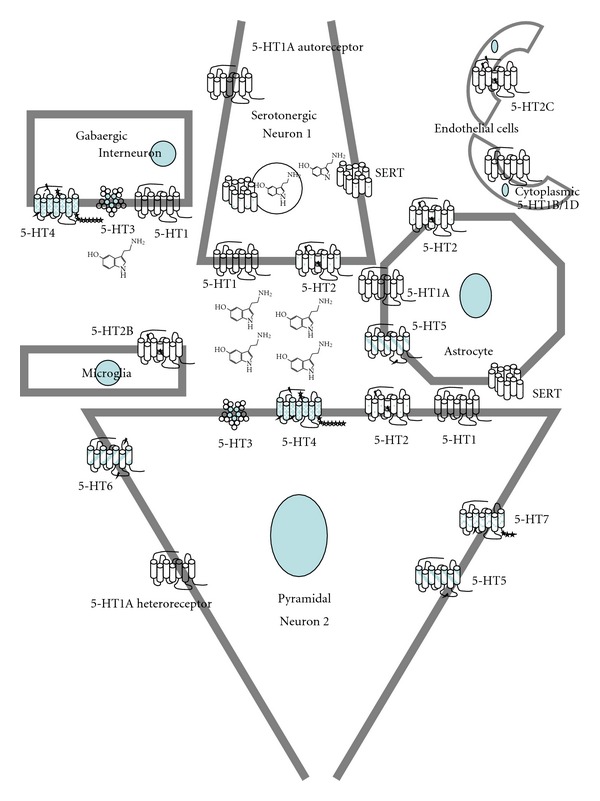
Serotonin receptors in hippocampus. The functional glia-neuron-vascular cells network uses several serotonin receptors (5-HTRs). The 7TMD images of each subtype receptor are represented with the defined number of exons that code for the mature protein (Bockaert et al., 2006) [44]; putative intron location in correspondent pre-mRNA is marked by a lightning symbol (*↯*); alternative splicing sites are marked with stars (*⋆⋆⋆*). Neuron metabotropic 5-HTRs are mainly somatodendritic volume receptors although there is an association with synaptic specializations for some of them. 5-HT_3_ with the five 4TMD subunits of a ligand activated ion channel is shown as synaptic receptor although this fact remains to be determined in hippocampus. Microglia is also included in the network for its relevance in pathophysiological responses, with 5-HT_2B_ receptor expression (Capone et al., 2007) [90]. The 12TMD image of the serotonin transporter (SERT; 5-HTT) and vesicular monoamine transporter (VMAT) are represented in the serotonergic neuron and only SERT in the astrocyte.

Serotonergic neuronal-glial interactions (Figure 6) have been proposed to play a significant role in the development of several CNS pathologies [163]. Some serotonin receptors are mainly expressed in glia. 5-HT_5A_ correlates with astrocyte maturity and activity, increasing its levels after induced gliosis [120] although its expression in pyramidal cells of hippocampus has been reported [117]. Addition of cAMP analogues to astrocyte cultures decreases 5-HT_1A_ expression and increases 5-HT_5A_, therefore suggesting a direct neuronal regulation of astrocyte homeostasis, as cAMP intracellular increases might activate and sensitize astrocytes to respond at serotonin signaling from neurons that can supress gliosis *in vivo* [120].

Each cell type can modify its serotonin receptor expression depending on the differentiation time and relationship in a particular network. Mouillet-Richard et al. (2000) have shown the differentiating changes than inducted serotonergic 1C11^∗5HT^ cells can exhibit [164], sequentially expressing three different serotonin receptor subtypes (5-HT_1B/1D_, 5-HT_2B_, and 5-HT_2A_). Although cell cultures do not represent reliable conditions of *in vivo* differentiation, they help us understand how cells can adapt to changing media. The 5-HT_2_ receptors are referred to as programmable receptors that may not influence development although this process affect their number, affinity, or function; the coupling efficiency of the receptor may change in time, in correlation to a developmental change of phosphatidylinositol hydrolysis-second messenger system [165].

In conclusion, the specific changes that modulate serotonin signaling can be performed by serotonin itself; the levels of serotonin that can be reached in the synapses, or as a volume transmission, is of outstanding importance to understand the rate of change in the 5-HT signaling itself, time of action might conduce to one response or the contrary, considering that all the cell types in hippocampus are involved in this modulation and function. Serotonin can act directly into neuron and glia after SERT incorporation, an ancient function for this biogenic amine and probably with more importance during development.
